# Machine Learning Approaches for Predicting Acute Respiratory Failure, Ventilator Dependence, and Mortality in Chronic Obstructive Pulmonary Disease

**DOI:** 10.3390/diagnostics11122396

**Published:** 2021-12-20

**Authors:** Kuang-Ming Liao, Chung-Feng Liu, Chia-Jung Chen, Yu-Ting Shen

**Affiliations:** 1 Department of Internal Medicine, Chi Mei Medical Center, Chiali 72263, Taiwan; abc8870@yahoo.com.tw; 2 Department of Medical Research, Chi Mei Medical Center, Tainan 71004, Taiwan; ting1992yu@gmail.com; 3 Department of Information Systems, Chi Mei Medical Center, Tainan 71004, Taiwan

**Keywords:** chronic obstructive pulmonary disease, machine learning, prediction model, acute respiratory failure, ventilator dependence, mortality

## Abstract

Chronic obstructive pulmonary disease (COPD) is one of the leading causes of mortality and contributes to high morbidity worldwide. Patients with COPD have a higher risk for acute respiratory failure, ventilator dependence, and mortality after hospitalization compared with the general population. Accurate and early risk detection will provide more information for early management and better decision making. This study aimed to build prediction models using patients’ characteristics, laboratory data, and comorbidities for early detection of acute respiratory failure, ventilator dependence, and mortality in patients with COPD after hospitalization. We retrospectively collected the electronic medical records of 5061 patients with COPD in three hospitals of the Chi Mei Medical Group, Taiwan. After data cleaning, we built three prediction models for acute respiratory failure, ventilator dependence, and mortality using seven machine learning algorithms. Based on the AUC value, the best model for mortality was built by the XGBoost algorithm (AUC = 0.817), the best model for acute respiratory failure was built by random forest algorithm (AUC = 0.804), while the best model for ventilator dependence was built by LightGBM algorithm (AUC = 0.809). A web service application was implemented with the best models and integrated into the existing hospital information system for physician’s trials and evaluations. Our machine learning models exhibit excellent predictive quality and can therefore provide physicians with a useful decision-making reference for the adverse prognosis of COPD patients.

## 1. Introduction

Chronic obstructive pulmonary disease (COPD) is a long-term, systemic inflammatory disease. It leads to the destruction of the lung parenchyma, small airway inflammation, and fibrotic changes [1]. After the lung tissue becomes damaged and scarred, pulmonary fibrosis ensues, resulting in irreversible airflow and airway obstructions even after bronchodilator treatment [2]. COPD is the third leading cause of death in the world with 3.23 million mortalities in 2019 alone [3]. One of the most important treatment goals for patients with COPD is to reduce the occurrence of acute exacerbations [4]. Frequent acute exacerbation results in the rapid decline of lung function [5], leading to frequent hospital admission, acceleration of disease progression, acute respiratory failure, ventilator dependence, and increased risk for mortality [6]. Previous studies used different factors to predict acute respiratory failure [7], ventilator dependence [8], and mortality [9].

Many healthcare systems and design practitioners need an evidence-based approach to facilitate treatment planning and decision making [10]. Artificial intelligence and machine learning improving access to care, increasing accuracy, decreasing cost, and providing the greatest value enable physicians more efficiency to treat patients [11,12].

Over the past years, artificial intelligence and machine learning approaches have been acceleratingly applied in medicine and the health care system. Machine learning approaches can improve care allocation and risk prediction in breast cancer [13], stroke imaging [14], heart failure diagnosis, classification [15], and readmission risk [16] and assessment of coronary artery disease in cardiac computed tomography [17]. Computer science is drastically improving and being combined with new algorithms based on neural network methods, allowing enormous progress in the development of machines capable of performing tasks for disease research and prediction [18]. Previous studies used artificial intelligence for the respiratory medicine [19,20,21,22,23] and diagnosis of COPD [24], while some looked into environmental factors, lifestyle data, and symptoms for the early detection of acute exacerbation in COPD [25]. However, there is currently no study that investigated the possibility of having acute respiratory failure, ventilator dependence, and mortality after hospitalization in patients with COPD using modern artificial intelligence and machine learning. Our study aims to characterize high-risk COPD patient groups and identify the factors that potentially increase the risk for acute respiratory failure, ventilator dependence, and mortality using machine learning for patients with COPD.

## 2. Materials and Methods

### 2.1. Study Design, Setting, and Samples

**Figure 1:**
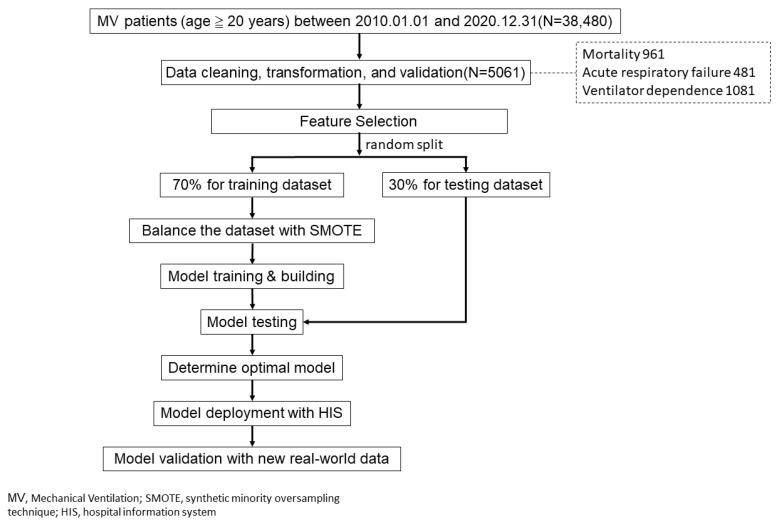
Research flow.

This retrospective study collected the data of patients with COPD (pulmonary obstruction) with either emergency, outpatient, or inpatient orders from the three hospitals of Chi Mei Medical Group in Taiwan (1 medical center, 1 regional hospital, and 1 district hospital), from 1 January 2010 to 31 December 2019, whose first six diagnosis codes are ICD-9: 490, 491,492, 496 or ICD-10: J41, J42, J43, J44. Data of patients who were less than 20 years old at the time of diagnosis and those with incomplete records, missing values, and ambiguous values were excluded from the study. Overall, data from 5061 patients were included for the predictive model analysis (Figure 1).

### 2.2. Feature and Outcome Variables

We chose 3 outcome variables to establish the prediction models: (1) mortality (in-hospital), (2) acute respiratory failure (in-hospital), and (3) ventilator dependence (continuous use of a respirator for 21 days during hospitalization).

Furthermore, based on literature evidence and clinical experience, this study included multiple factors (features) of demographics and clinical information that affect the three outcome variables, including age, gender, BMI, vital signs of body temperature (BT), pulse, Glasgow Coma Scale (GCS) and respiratory rate (RR; the worst record in-hospital), SPO2 (the worst record in-hospital), WBC, Hb, platelet, BUN, CRP, Na, K, PH, Pao2, Paco2, Hco3, and presence of comorbidities (e.g., diabetes, hypertension, pneumonia, etc.).

### 2.3. Model Building and Evaluation

For maximizing model performance, we used all the variables (28, usually available in clinical) to build our prediction models without performing any feature selection process in advance. The data were randomly stratified into a training dataset for model building (70%) and a testing dataset for model validation (30%). The overall research process is shown in Figure 1. Because there were fewer positive classes (outcomes to be predicted such as death, etc.) in the clinic, the SMOTE method (synthetic minority over-sampling technique) [26] was used to improve the data imbalance in the training dataset. Each outcome was paired with 7 machine learning algorithms to build the predictive models. These algorithms include: (1) logistic regression, (2) random forest, (3) Support Vector Machine (SVM), (4) K-nearest neighbor (KNN), (5) LightGBM, (6) XGBoost, and (7) multilayer perceptron (MLP). Python and Scikit.learn machine learning tools were used.

Grid search with 5-fold cross-validation was used for tuning hyperparameters to build the best models based on the training dataset. Hyperparameter tuning was conducted by giving ranges of specific hyperparameter values manually, such as {num_iterations: (100, 1000), max_depth: (–1,4), learning_rate: (0, 0.001), feature_fraction: (0.6, 1), num_leaves: (10, 31)} for LightGBM model, {n_estimators: (100, 200, 500, 700), max_features: (‘auto’, ‘sqrt’, ‘log2’), max_depth: (5, 6, 7, ’None’), criterion: (‘gini’)} for Random Forest model.

After a model was built, we then used the testing dataset to validate the models with well-defined model performance indicators of accuracy, sensitivity, specificity, and AUC (area under the receiver operating characteristic curve). The model with the highest AUC value was regarded as the best model and exported as model file (PKL file in Python) for further implementing a prediction system for practical use.

## 3. Results

**Figure 2:**
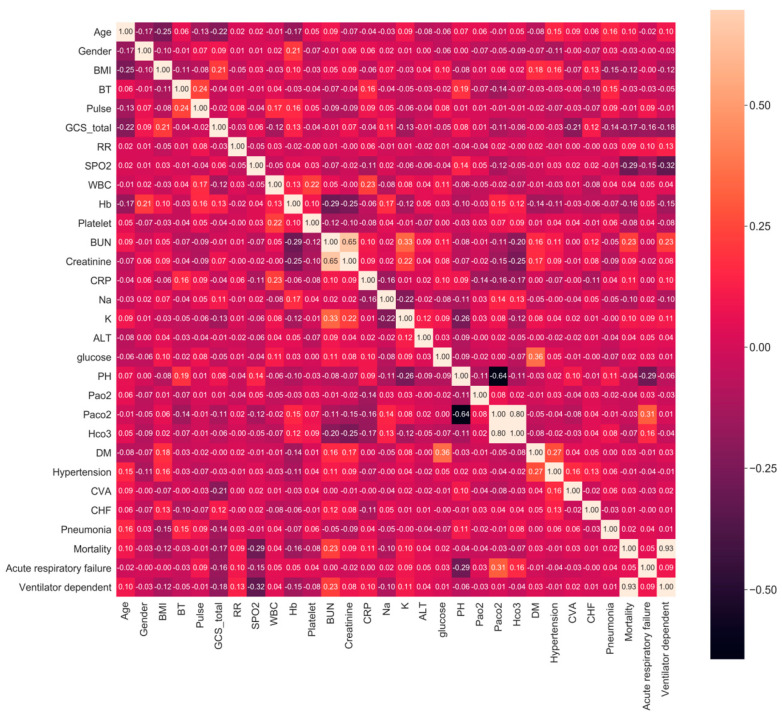
Spearman correlation.

There were 38,480 raw samples collected; after applying the exclusion criteria, a total of 5061 were used for model building and analysis. Table 1 shows that the mean age of patients was 77 years old and that 67% were males while 33% were females. Spearman correlation analysis (see Figure 2) identifies the correlation between the outcome and each feature. For mortality and ventilator dependence, the most relevant features were SPO2, BUN, and GCS, while for acute respiratory failure, the most relevant features were Paco2, PH, and GCS.

**Figure 3:**
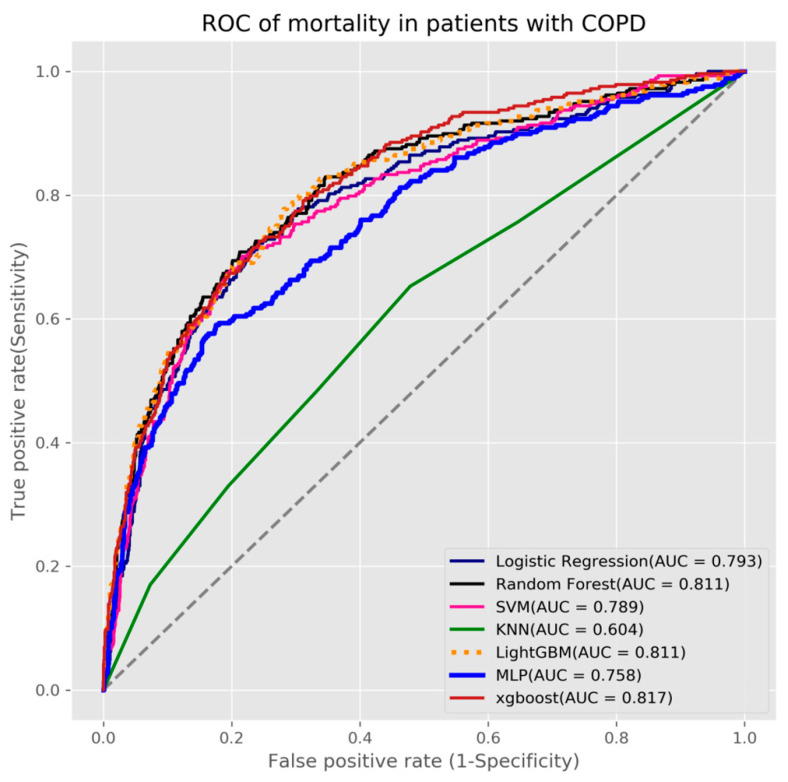
ROC of mortality in patients with COPD.

**Figure 4:**
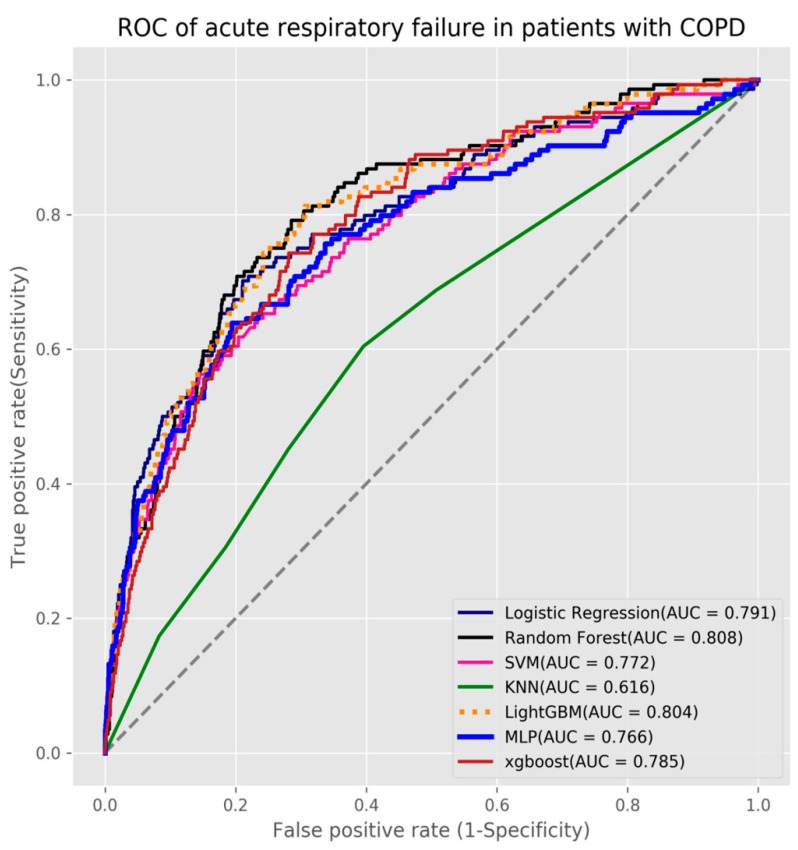
ROC of acute respiratory failure in patients with COPD.

**Figure 5:**
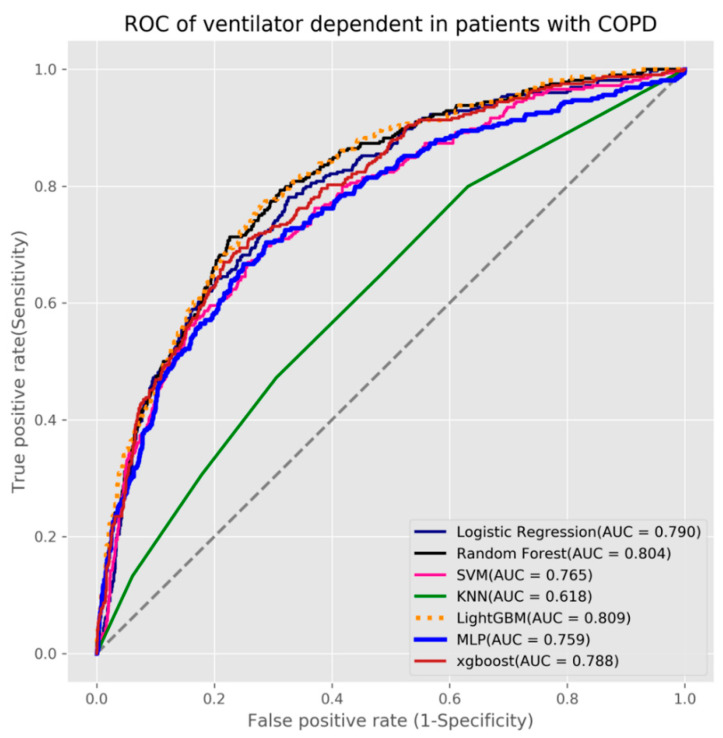
ROC of ventilator dependence in patients with COPD.

In this study, three outcome prediction models were established for predicting mortality, acute respiratory failure, and ventilator dependence. We used seven machine learning algorithms to build the three models. We used grid search with 5-fold cross-validation for each algorithm to obtain the best hyperparameters and build the final production models. The results showed that the XGBoost algorithm obtained the highest AUC value (0.817) for mortality as its prediction model (see Table 2 and Figure 3), the random forest algorithm had the highest AUC value (0.804) for acute respiratory failure (see Table 3 and Figure 4), and LightGBM algorithm had the highest AUC value (0.809) for ventilator dependence (see Table 4 and Figure 5).

**Figure 6:**
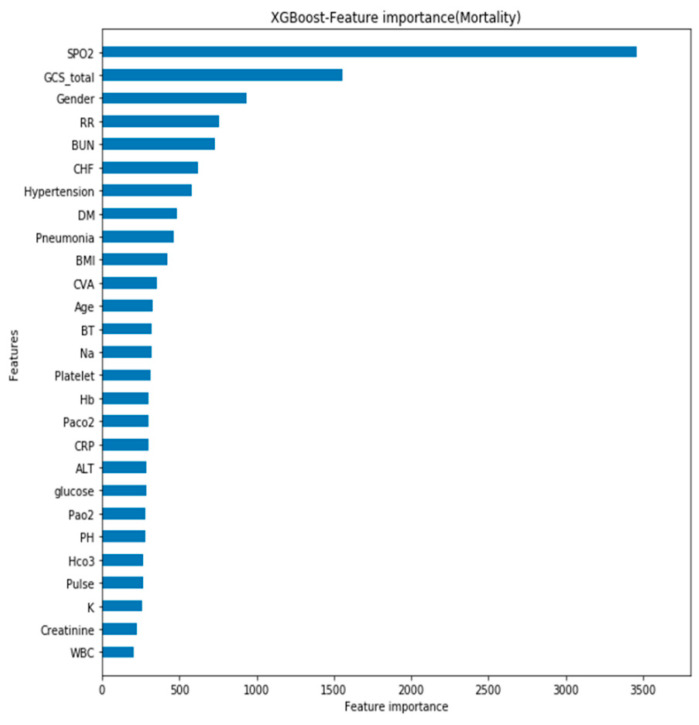
Feature importance of mortality.

**Figure 7:**
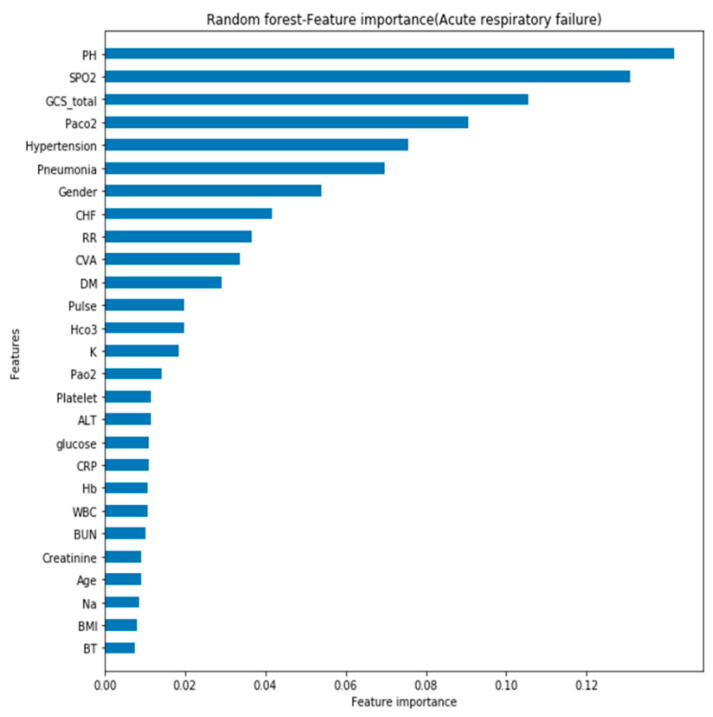
Feature importance of acute respiratory failure.

**Figure 8:**
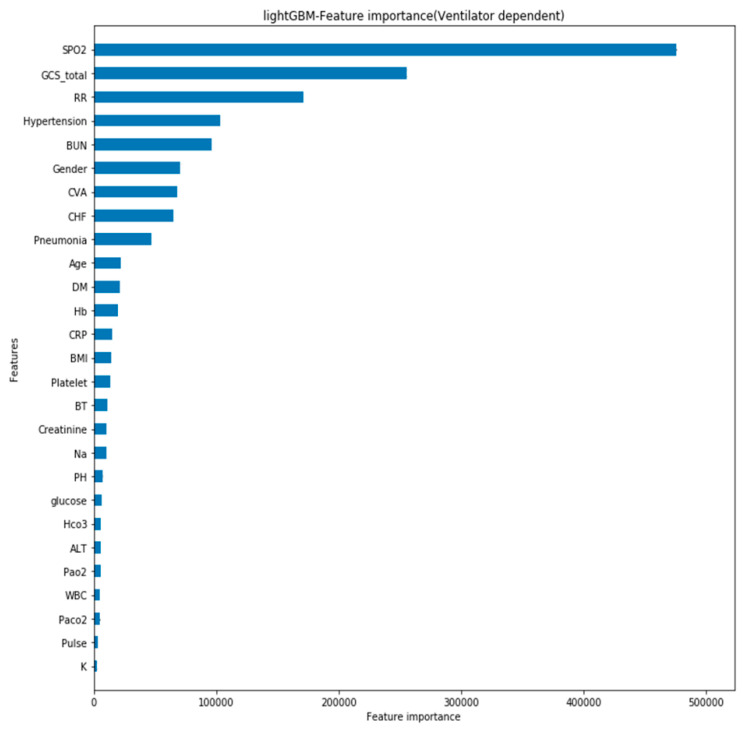
Feature importance of ventilator dependence.

In addition, feature importance graph refers to techniques that assign a score to input features based on how useful they are at predicting a target variable in graphic form. Thus, during the model training process, the feature importance graph with criteria of information gain was utilized in Python to judge the importance of each feature to the model. It showed that the SPO2, GCS, and gender were the three most important features of the mortality model (see Figure 6); PH, SPO2, and GCS were the most important features of the acute respiratory failure model (see Figure 7); and SPO2, GCS, and RR were the most important features of the ventilator dependence model (see Figure 8).

**Figure 9:**
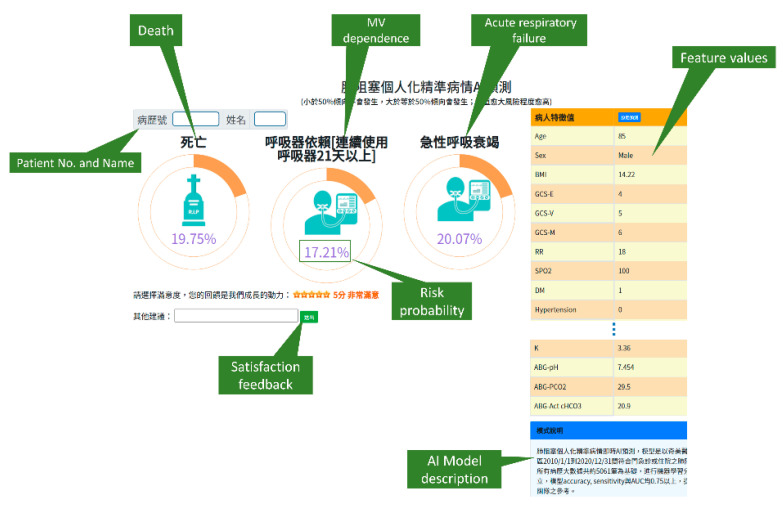
A snapshot of AI web service application for predicting outcomes of in-hospital COPD patients.

To verify the usefulness and feasibility of our models in clinical, we asked the hospital’s information system department to design a web service application with our best models and integrate it into the existing hospital information system (HIS, here, is inpatient physician ordering system). The predictive models were implemented in Python language, while the web service was implemented in MS Visual Studio .NET tool. Figure 9 shows the snapshot of the web service application.

Once a COPD patient is hospitalized, the physician can press the “AI button” in the existing inpatient physician ordering system when they need AI assistance, and a risk prediction webpage of the patient will automatically appear (Figure 9) without the need to manually enter patient information (features). The webpage graphically displays the risk probabilities of death, MV dependence, and acute respiratory failure; the probability greater than or equal to 50% means the risk tends to occur while less than 50% means tends not to occur—the greater the probability, the higher the risk. As shown in Figure 9, the AI predicts the patient will not die or have MV dependence and acute respiratory failure while hospitalized because the risks are all below 50%.

We demonstrated this AI web service application to three thoracic physicians for evaluation and trial. They all gave a high degree of acceptance relating to the graphic interface and reasonable risk values and believed that it was a significant help for clinical decision making.

## 4. Discussion

The features in this study included the most demographic data and clinical information, such as body mass index, gender, age, blood pressure, body temperature (BT), pulse, respiratory rate (RR), oxygen saturation level, GCS, and laboratory data (i.e., white blood cell, hemoglobin, platelet, blood urea nitrogen, creatinine, C-reactive protein, sodium, potassium, Alanine transaminase, glucose, PH, PaO2, PaCO2, and HCO3). We also included common and important comorbidities such as diabetes, hypertension, cardiovascular disease, congestive heart failure, and pneumonia. All of the mentioned features were employed to predict the outcome models (in-hospital mortality, acute respiratory failure during hospitalization, and ventilator dependence). These features are commonly used in clinical practice and are important markers in a patient’s health; thus, there was no need for the physician to deliberately arrange the examination. Moreover, this study analyzed systemic diseases and applied them to predict the prognosis of patients with COPD.

To our knowledge, this is one of few studies that used machine learning and big data techniques to practically predict the likelihood of patients with COPD to acquire acute respiratory failure, become ventilator dependent, and have increased chances for mortality after hospitalization. This study was the first to analyze the daily available data on patients with COPD from the system of three Chi Mei hospitals, including the medical center, regional hospital, and community hospital using different machine learning approaches (i.e., logistic regression, random forest, SVM, KNN, LightGBM, ML, and XGBoost) to select the best models to predict a patient’s outcome (i.e., acute respiratory failure, ventilator dependence, and mortality). The performance of each model was assessed using sensitivity, specificity, and AUROC metrics. This is by far the most comprehensive study that used machine learning models to predict COPD outcomes.

Goto et al. [27] used the National Hospital and Ambulatory Medical Care Survey ED data to identify patients with COPD exacerbation. They employed routinely available triage data as predictors (e.g., patient characteristic, arrival mode, vital signs, chief complaint, comorbidities, etc.) and four machine learning-based models (i.e., Lasso regression, random forest, boosting, and deep neural network) and compared them with traditional logistic regression. In addition to patient characteristics, vital signs, and comorbidities, our study added more laboratory data, utilized seven machine learning-based models, and focused on acute respiratory failure, ventilator dependence, and mortality as outcomes.

Peng and colleagues [28] used C5.0 decision tree classifier to predict the prognosis of patients with COPD with acute exacerbation. Their overall accuracy was 80.3%, with 95% CI (0.6991, 0.8827) and Kappa of 0.6054. The models established in this study had an accuracy of more than 80%.

In addition, Shah et al. [29] recruited 110 patients and followed them for more than 35,000 days. They used a finite-state machine-based approach to predict the acute exacerbation of COPD during home monitoring. They found that vital signs obtained from a pulse oximeter (i.e., respiratory rate, pulse rate, and oxygen saturation) could predict exacerbation events and that oxygen saturation was more predictive than respiratory rate and pulse rate. Our study also integrated these vital signs and the results showed that they improved the positive predictive accuracy of the machine learning. We summarized the comparison of these works in Table 5.

Previous studies mostly focused on acute exacerbation of COPD [30,31,32] and little attention has been paid to the impact of acute respiratory failure, ventilator dependence, and mortality. All of these outcomes are important for patients, patient’s families, and the medical team. The lack of patient-centered care and early prediction of respiratory failure and mortality could contribute to poor outcomes, suboptimal use of medical resources, and deteriorated psychological sequelae of patients and family members [33]. In addition to appropriate diagnosis and management of COPD [34], it is important to determine the possible outcomes of patients with COPD after hospitalization as early as possible so that prompt and effective treatment could be given to improve their prognosis. One of the most important tasks of a physician is to inform the patient and their loved ones about the seriousness of their condition [35]. The information may alter a patient’s view of their future and cause additional stress [36]. Therefore, more precise and early detection methods are needed to ensure that the information about a patient’s prognosis is as accurate as possible so as not to add more stress and anxiety to the patient and their family [37].

The current study used common and important features to predict COPD prognosis. Further, the machine learning techniques that we established could provide physicians an opportunity to develop algorithms that integrated complex interaction factors to offer different possible prognoses to patients with COPD [38]. The addition of patient demographics, laboratory data, and comorbidities in this study to predict the possible outcomes of patients with COPD was successfully modeled. From the optimal models, we were also able to identify the important features that could affect the patients’ outcomes. The results indicated that SpO2 and GCS were the most important features for acute respiratory failure and ventilator dependence with the addition of gender for mortality; thus, compared with other features, they are the most crucial outcome predictors.

Our study also has some limitations that need to be addressed. First, although our data included different hospital levels, from a medical center to a regional hospital, to represent different disease severity, these only came from three hospitals in Taiwan. Future studies could include more hospitals for a more representative sample. Second, the models were constructed based on the Asian population with COPD; thus, it may not be accurate for the Caucasian population. Third, smoking history and duration were considered important risk factors in COPD prognosis; however, this study was not able to include them because of failure to accurately retrieve this information from the electronic health record.

## 5. Conclusions

To build a generic machine model to help physicians and support their diagnosis of disease progression and risk of death for patients with COPD, this study developed a machine learning classifier using patients’ features such as basic health indicators, comorbidity indicators, and inflammatory indicators. We also implemented and integrated a web-based predictive application into the existing HIS and obtained high acceptance from physicians after initial use. We believe that predicting the adverse outcomes of patients with COPD using machine learning algorithms is a promising research approach to help physicians assess the severity of the disease after hospital admission at the earliest possible time. These could guide them in choosing the most appropriate treatment strategies to improve the prognosis of their patients. For follow-up study, researchers can include more potential variables and perform a feature selection process to improve the quality of the models.

## Figures and Tables

**Table 1:** Demographics.

Feature	Overall	Mortality		*p*-Value	Acute Respiratory Failure		*p*-Value	Ventilator Dependence		*p*-Value
		No	Yes		No	Yes		No	Yes	
	5061	4100	961		4580	481		3980	1081	
Age, mean (SD)	77.8 (11.4)	77.3 (11.4)	80.2 (11.2)	<0.001	77.9 (11.3)	77.1 (12.2)	0.159	77.2 (11.3)	79.9 (11.4)	<0.001
Sex_female, n (%)	1673 (33.1)	1326 (32.3)	347 (36.1)	0.028	1512 (33.0)	161 (33.5)	0.879	1289 (32.4)	384 (35.5)	0.057
Sex_male, n (%)	3388 (66.9)	2774 (67.7)	614 (63.9)		3068 (67.0)	320 (66.5)		2691 (67.6)	697 (64.5)	
BMI, mean (SD)	23.5 (5.4)	23.8 (5.6)	22.1 (4.5)	<0.001	23.5 (5.4)	23.4 (6.1)	0.756	23.8 (5.5)	22.3 (4.8)	<0.001
BT, mean (SD)	37.1 (1.1)	37.1 (1.1)	37.0 (1.1)	0.039	37.1 (1.1)	37.0 (1.1)	0.024	37.1 (1.1)	37.0 (1.1)	0.001
Pulse, mean (SD)	101.9 (23.8)	102.0 (22.9)	101.4 (27.3)	0.529	101.2 (23.5)	108.5 (26.0)	<0.001	102.0 (22.8)	101.5 (27.3)	0.648
GCS, mean (SD)	13.2 (3.1)	13.4 (2.9)	12.1 (3.7)	<0.001	13.4 (2.9)	11.6 (4.2)	<0.001	13.5 (2.9)	12.1 (3.7)	<0.001
RR, mean (SD)	21.7 (6.0)	21.4 (5.5)	22.8 (7.8)	<0.001	21.5 (5.5)	23.5 (9.3)	<0.001	21.3 (5.0)	23.2 (8.7)	<0.001
SPO2, mean (SD)	84.9 (16.9)	87.3 (14.6)	74.9 (21.7)	<0.001	85.8 (16.0)	77.2 (22.7)	<0.001	87.8 (13.8)	74.5 (22.2)	<0.001
Lab data										
WBC, mean (SD)	10.3 (4.8)	10.2 (4.7)	10.7 (5.1)	0.01	10.3 (4.8)	11.1 (5.2)	0.001	10.2 (4.8)	10.7 (5.0)	0.003
Hb, mean (SD)	12.1 (2.4)	12.3 (2.4)	11.3 (2.5)	<0.001	12.1 (2.4)	12.5 (2.6)	0.001	12.3 (2.4)	11.4 (2.5)	<0.001
Platelet, mean (SD)	174.1 (49.3)	176.1 (47.7)	165.6 (54.5)	<0.001	173.5 (49.6)	179.7 (46.1)	0.006	176.1 (47.7)	167.0 (54.2)	<0.001
BUN, mean (SD)	28.4 (18.0)	26.4 (16.4)	37.0 (21.7)	<0.001	28.4 (18.1)	28.5 (16.9)	0.952	26.3 (16.3)	36.3 (21.6)	<0.001
Creatinine, mean (SD)	1.5 (1.3)	1.4 (1.2)	1.7 (1.5)	<0.001	1.5 (1.3)	1.4 (1.2)	0.173	1.4 (1.2)	1.7 (1.5)	<0.001
CRP, mean (SD)	53.5 (63.9)	50.2 (62.4)	67.5 (68.3)	<0.001	53.5 (63.7)	53.7 (66.0)	0.958	50.0 (62.4)	66.4 (67.7)	<0.001
Na, mean (SD)	135.2 (6.9)	135.5 (6.4)	133.7 (8.6)	<0.001	135.1 (6.9)	135.6 (7.6)	0.232	135.5 (6.4)	133.8 (8.5)	<0.001
K, mean (SD)	3.96 (0.69)	3.92 (0.67)	4.11 (0.77)	<0.001	3.94 (0.68)	4.15 (0.76)	<0.001	3.92 (0.66)	4.10 (0.78)	<0.001
ALT, mean (SD)	42.3 (138.7)	39.7 (132.8)	53.5 (161.0)	0.014	40.2 (114.1)	62.1 (279.5)	0.089	39.4 (133.5)	53.2 (155.9)	0.008
Glucose, mean (SD)	166.3 (86.2)	165.6 (85.7)	169.2 (88.6)	0.253	165.3 (87.3)	175.2 (75.3)	0.007	165.6 (85.8)	168.7 (87.8)	0.3
PH, mean (SD)	7.4 (0.1)	7.4 (0.1)	7.4 (0.1)	0.018	7.4 (0.1)	7.3 (0.1)	<0.001	7.4 (0.1)	7.4 (0.1)	<0.001
Pao2, mean (SD)	139.4 (78.5)	140.9 (78.2)	133.1 (79.5)	0.006	138.6 (76.9)	147.2 (92.0)	0.049	140.6 (77.6)	135.1 (81.5)	0.045
Paco2, mean (SD)	40.0 (16.8)	40.2 (16.7)	38.9 (17.5)	0.036	38.3 (14.1)	56.1 (28.3)	<0.001	39.9 (16.2)	40.3 (19.0)	0.517
Hco3, mean (SD)	24.4 (6.6)	24.6 (6.5)	23.4 (7.1)	<0.001	24.0 (6.2)	27.7 (9.0)	<0.001	24.5 (6.4)	23.9 (7.4)	0.006
Comorbidity										
DM, n (%)	1775 (35.1)	1412 (34.4)	363 (37.8)	0.056	1614 (35.2)	161 (33.5)	0.47	1366 (34.3)	409 (37.8)	0.035
Hypertension, n (%)	2920 (57.7)	2375 (57.9)	545 (56.7)	0.516	2670 (58.3)	250 (52.0)	0.009	2308 (58.0)	612 (56.6)	0.437
CVA, n (%)	839 (16.6)	657 (16.0)	182 (18.9)	0.032	774 (16.9)	65 (13.5)	0.066	642 (16.1)	197 (18.2)	0.111
CHF, n (%)	1293 (25.5)	1035 (25.2)	258 (26.8)	0.325	1173 (25.6)	120 (24.9)	0.793	1009 (25.4)	284 (26.3)	0.565
Pneumonia, n (%)	3251 (64.2)	2617 (63.8)	634 (66.0)	0.226	2915 (63.6)	336 (69.9)	0.008	2542 (63.9)	709 (65.6)	0.313

Note: SD, standard deviation.

**Table 2:** Testing results of the predictive models for mortality.

Algorithm	Accuracy	Sensitivity	Specificity	AUC
Logistic Regression	0.733	0.733	0.733	0.793
Random Forest	0.735	0.736	0.734	0.811
SVM	0.768	0.691	0.786	0.789
KNN	0.633	0.483	0.668	0.604
LightGBM	0.744	0.743	0.744	0.811
MLP	0.683	0.681	0.683	0.758
XGBoost	0.727	0.733	0.726	0.817

**Table 3:** Testing results of the predictive models for acute respiratory failure.

Algorithm	Accuracy	Sensitivity	Specificity	AUC
Logistic Regression	0.738	0.736	0.738	0.791
Random Forest	0.747	0.75	0.747	0.812
SVM	0.784	0.604	0.803	0.772
KNN	0.694	0.451	0.719	0.616
LightGBM	0.756	0.75	0.756	0.804
MLP	0.71	0.708	0.71	0.766
XGBoost	0.723	0.722	0.723	0.785

**Table 4:** Testing results of the predictive models for ventilator dependence.

Algorithm	Accuracy	Sensitivity	Specificity	AUC
Logistic Regression	0.72	0.719	0.72	0.79
Random Forest	0.733	0.735	0.733	0.803
SVM	0.755	0.596	0.798	0.765
KNN	0.647	0.472	0.695	0.618
LightGBM	0.739	0.738	0.739	0.809
MLP	0.699	0.704	0.698	0.759
XGBoost	0.724	0.719	0.725	0.788

**Table 5:** A comparison with related studies.

Study	This Study	[27]	[28]	[29]
Patient type	Inpatient COPD	Emergency department, Asthma or COPD exacerbation	Inpatient AECOPD	COPD at home
Patient number	5061	3206	410	110
Outcome	1. Ventilator dependence 2. Respiratory failure 3. Mortality	1. Critical care outcome 2. Hospitalization outcome	Classifying the severity of AECOPD	Predicting COPD exacerbations
Study method	Seven machine leaning methods	Four machine leaning methods	Four machine leaning methods	One machine leaning method
Real world implementation	Yes. A predictive application with AI models was implemented and integrated into the existing HIS	N/A	N/A	N/A.
Input data	Patient demographic, vital signs, Glasgow Coma Scale (GCS), blood gases, laboratory results, comorbidities	Age, sex, mode of arrival, vital signs, common chief complaints, asthma or COPD status, comorbidities	Vital signs, medical history, comorbidities, various inflammatory indicators, laboratory results	Vital signs
Testing results (AUC)	Ventilator dependence (0.618–0.809)	Critical care outcome (0.76–0.80)	Predicting the prognosis (0.667–0.803)	Predicting COPD exacerbations (0.682)
	Acute respiratory failure (0.616–0.812)	Hospitalization outcome (0.82–0.83)		
	Mortality (0.604–0.817)			
Year	2021	2018	2020	2017

## Data Availability

The dataset used for this study is available on request to the corresponding author.
